# Heavy Metal Contamination Near Industrial Estate Areas in Phra Nakhon Si Ayutthaya Province, Thailand and Human Health Risk Assessment

**DOI:** 10.3390/ijerph15091890

**Published:** 2018-08-31

**Authors:** Rachaneekorn Mingkhwan, Suwalee Worakhunpiset

**Affiliations:** Department of Social and Environmental Medicine, Faculty of Tropical Medicine, Mahidol University, 420/6 Ratchavithi Rd, Bangkok 10400, Thailand; Ruchneekorn.min@mahidol.ac.th

**Keywords:** heavy metals, industrial estate, ayutthaya, risk assessment, hazard quotient, hazard index

## Abstract

Industrial activity is one of the significant sources of environmental contamination with heavy metals, especially in developing countries. Flood can also lead to the distribution of toxic substances into the environment, regarding the Thailand flood in 2011 as some industrial estates are affected, leading to concern about heavy metals from industrial wastewater contamination. We aimed to measure the levels of Cd, Cr, Cu, Ni, Mn, Pb, and Zn in river and stream water, sediment, and fish collected from the area around the industrial estates in Uthai District and Bangpa-in District of Phra Nakhon Si Ayutthaya Province, following the floods of 2011. The results revealed that heavy metal levels in water did not exceed Thailand surface water quality standards, except for Mn levels at one sampling site. Metal levels in sediment and fish samples also did not exceed published standards. The hazard quotient for fish consumption was highest for Ni (0.2178) in *Trichopodus trichopterus* collected from the area near the industrial estate in Bangpa-in District, while the hazard index from Cd, Cr, and Cu exposure were 0.86966, which was lower than 1, indicating that the health risks for these seven metals were within acceptable ranges.

## 1. Introduction

Thailand’s socio-economic structure has been changing rapidly from an agricultural to an industrial society. The increase in industrial activities has contributed to environmental pollution throughout the country, raising health concerns. The Chao Phraya and Pasak Rivers are considered major water sources in Chao Phraya River basin, Thailand. Over 30,000 industrial facilities located in the Chao Phraya River basin have contributed to river water contamination by toxic substances, including heavy metals from industrial effluents [1]. In addition, flooding is one extreme event that can play a significant role in toxic substance dispersion to the environment. Regarding the Thailand catastrophic flooding in 2011, industrial estates in central Thailand were flooded, raising concern about toxic chemical contamination from wastewater treatment systems of industrial estates in Phra Nakhon Si Ayutthaya Province [2]. Moreover, the Thai News Agency also reported relevant water pollution at the mouth of the Pratunam Canal in Uthai District, Phra Nakhon Si Ayutthaya Province in 2013 [3]. The purpose of this study was to determine the levels of heavy metals in water, sediment, and aquatic organisms, especially edible fish, in the area near flood-affected industrial estates in Phra Nakhon Si Ayutthaya Province. The health risk posed by consuming fish caught in the area was also evaluated.

## 2. Materials and Methods

### 2.1. Study Sites

**Figure 1:**
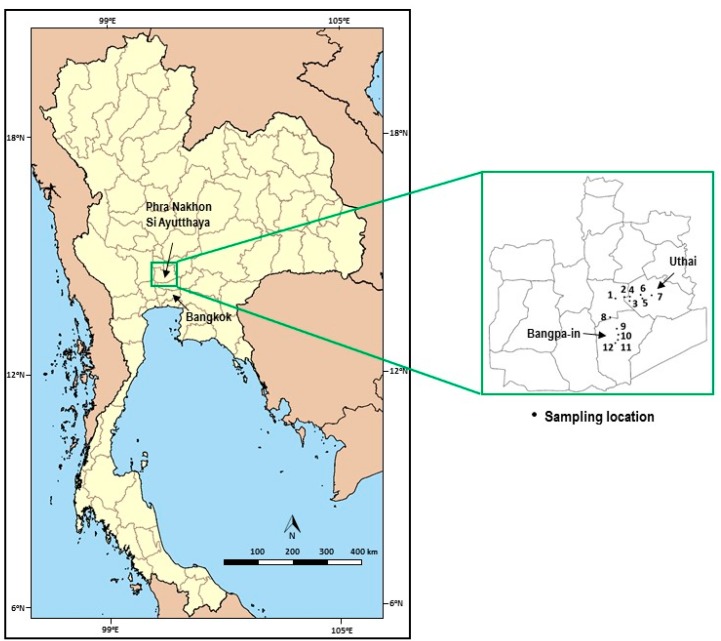
Map of study sites and sampling locations.

The study sites were located in Phra Nakhon Si Ayutthaya Province, including the area near industrial estates in Uthai District and Bangpa-in District, approximately 67 km from Bangkok (Figure 1). The industrial estates in these study sites focused on automotive; electronics industry and electric appliances; metal products, machinery, and transport equipment; chemical, paper, and plastics; and office automation industries.

### 2.2. Sample Collection

Surface water, including river and stream water, sediment, and fish samples were collected from 7 locations in Uthai District and 5 locations in Bangpa-in District, representing reference points, industrial effluent receiving areas, and downstream areas on three separate occasions in January, May, and August 2014, corresponding to the cold, summer, and rainy seasons. The sampling locations ranged between Latitude 14°14′13.00″ N–14°21′40.33″ N, Longitude 100°34′0.35″ E–100°41′53.91″ E.

One kilogram of sediment was collected at a vertical depth of 6 inches using a shovel. Sediment samples were kept in sealed polyethylene bags and stored at 4 °C until analysis [4].

Fish were collected using a net, and represent the species present at the study sites. A total of eleven fish species were collected at the study sites near the industrial estate in Uthai District and Bangpa-in District, and each species was collected in a polyethylene bag and stored at −70 °C until analysis [5].

Surface water samples were collected in 1-L polyethylene bottles, preserved at pH < 2, and stored at 4 °C until analysis. Water temperature, pH, dissolved oxygen, total suspended solids, and conductivity were measured on site using Hach Sension 156 and Sension 378 multiparameter meters (Loveland, CO, USA) [6,7].

### 2.3. Sample Preparation

Sediment sample: Sediment samples were dried in an oven at 110 ± 5 °C for 24 h to a constant weight [8] and prepared following US EPA method 3050B [9]. Briefly, 1 g of sediment sample was digested with repeated additions of concentrated nitric acid (analytical grade) at 95 ± 5 °C until completely digested, then filtered and made a final volume to 100 mL.

Fish sample: Muscular tissues of fish samples were carefully separated and dried in an oven at 110 ± 5 °C for 24 h to a constant weight [8]. The dried tissue samples were grinded using a mortar and pestle to fine, homogenized powder, then stored in polyethylene bags in desiccators until digestion following NIOSH method 8005 [10]. Briefly, 1 g of fish sample was digested with 5 mL of concentrated nitric acid (analytical grade) at 110 °C for 2 h, then filtered and made a final volume to 5 mL.

Water sample: Water samples were prepared by acid digestion following US EPA method 200.9 [11]. Briefly, 100 mL of water sample was digested with 2 mL (1 + 1) nitric acid (analytical grade) and 1 mL of (1 + 1) hydrochloric acid (analytical grade) at 85 °C for 2 for 2 h until the sample aliquot was reduced to about 20 mL, and made the final volume to 50 mL.

All digested samples were stored at 4 °C until analysis.

### 2.4. Analysis of Heavy Metals

Concentrations of Cd, Cr, Cu, Mn, Ni, Pb, and Zn in the digested samples were determined using a polarized Zeeman Atomic Absorption Spectrophotometer (Hitachi, Tokyo, Japan). Each sample was analyzed in duplicate. Quality control was performed by spiking the pooled fish sample with known concentrations of heavy metal standards (Merck, Kenilworth, NJ, USA) to attain the final concentration of 1.0 and 3.0 µg/g and digested with the same manner with the samples while the unspiked pooled fish samples were used as control. The analyzed amount of metal of the spiked samples was used to calculate percent recovery after corrected by the concentration measured in the control sample. The recovery percentages of the seven metals ranged from 90.10 to 109.66%; relative standard deviation was 0.09–10.95%. For every 10 samples the control was analyzed for accuracy checking. The analysis results of sediment and fish samples reported in dry weight (dw).

### 2.5. Statistical Analysis

Statistical analyses were performed using SPSS version 20.0 software (IBM, Armonk, NY, USA). After performing the Kolmogorov-Smirnov test, Mann-Whitney U test and Kruskal Wallis test were used to identify significant differences (*p* < 0.05) of heavy metal concentrations in water, sediment, and fish samples between study site and among season.

### 2.6. Fish Consumption by the Local Population

Fish consumption rates of the local population were estimated by using responses to questionnaires administered after ethical approval by the Ethical Committee of the Faculty of Tropical Medicine, Mahidol University (approval TMEC 14-017). One hundred individuals residing in the study areas for at least one year and aged over 18 years were randomly selected and invited to participate. After signing informed consent forms, participants were interviewed. The fish consumption rates reported by the local population varied by species, and ranged from 12.6 to 21.5 g/day.

### 2.7. Quantitative Health Risk Assessment

The non-cancer human health risks posed by heavy metal exposure from fish consumption were estimated using the U.S. EPA risk assessment model [12]. The uptake rate or chronic daily intake of heavy metals from fish consumption was calculated based on the assumption for the worst case scenario whether the local people eat individual fish species at the highest amounts (21.5 g/day; data from questionnaire interview) everyday using the following equation:

$$\mathrm{CDI}=\frac{C\times \mathrm{IR}\times \mathrm{EF}\times \mathrm{ED}}{\mathrm{BW}\times \mathrm{AT}}$$

where CDI is the chronic daily intake (mg/kg-day), C equals the concentration of the heavy metal in fish (mg/kg), IR is the fish intake rate (mg/day), EF is the exposure frequency (days/year), ED is the exposure duration (years), BW is body weight (kg), and AT is the average time (period over which exposure is averaged-days).

Then hazard quotient (HQ), which indicates the potential of non-cancer health effects, was calculated with the following formula:

$$\mathrm{HQ}=\frac{\mathrm{CDI}}{\mathrm{RfD}}$$

where RfD is the oral reference dose for the heavy metal of interest. The RfD of Cd, Cr, Mn, Ni, and Zn were 0.001, 0.003, 0.01, 0.14, 0.02, 0.3 mg/kg-day, respectively [13,14,15,16,17]. Whereas the RfD of Pb did not establish due to insufficient information [18], we calculated the HQ of Pb as previously described by Lui et al. [19]. In addition, the RfD of Cu did not establish, and ATSDR minimal risk level of Cu was used to calculate HQ of Cu [20].

The calculated HQ ≤ 1 means the exposed population is supposed to be safe or the risk is acceptable whereas HQ > 1 indicates significant non-cancer risk from exposure to each heavy metal [12].

In addition, as Cr, Cd, and Cu can exert adverse effects on the liver, kidney, and immune system, a cumulative hazard index (HI) was calculated using equation as follows:

HI = HQ_Cr_ + HQ_Cd_ + HQ_Cu_

If HI ≤ 1, the non-cancer risk can be considered to be acceptable, while HI > 1 indicates significant non-cancer risk from consuming fish contaminated with Cr, Cd, and Cu [12].

## 3. Results

### 3.1. General Characteristics of Water Samples

Water samples in the two study areas did not differ significantly. The mean water temperatures of water samples in Uthai District and Bangpa-in District sampling sites were 32.7 and 32.6 °C; pH values were 7.15 and 7.21; dissolved oxygen levels were 3.68 and 4.00 mg/L; conductivity was 978.36 and 636.39 µS/cm; and total suspended solids values were 488.65 and 318.35 mg/L, respectively. The findings indicated that water in the study sites could be classified as Class 3 surface water (moderately clean fresh surface water used for consumption that should be treated before using as defined in the Surface Water Quality Standards) [7].

### 3.2. Heavy Metals in Water Samples

Most metal levels in the water samples were consistent with Thailand Surface Water Quality Standard [7], with the exception of Mn levels in one sample collected near the industrial estate in Uthai District, which was higher than the standard. Table 1 displays the heavy metal concentrations in surface water collected from two Districts. The average concentrations of Cu, Ni, Mn, and Pb between the two study sites differed significantly at *p*-value = 0.01, 0.03, 0.04, and 0.04, respectively. In addition, Cr levels differed over sampling seasons (*p*-value = 0.02).

### 3.3. Heavy Metals in Sediment Samples

Heavy metal levels in sediment samples from the two study sites were mostly within the standard of Soil Quality Used for Living and Agriculture [21]. Table 2 displays the ranges measured for the metals. Among seven analyzed metals, only Ni concentrations showed significant difference (*p* = 0.04) between the study sites. 

### 3.4. Heavy Metal Concentrations in Fish Samples

Samples of nine fish species, including *Trichopodus trichopterus* (Pallas, 1770), *T. microlepis*, *Anabas testudineus* (Bloch, 1792), *Pristolepis fasciata*, *Channa striata*, *Oreochromis niloticus*, *Notopterus notopterus*, *Puntius brevis*, and *Puntioplites proctozysron* were collected at the study sites near the industrial estate in Uthai District (Table 3). For overall fish samples collected at this site, the average concentration of Cd, Cr, Cu, Mn, Ni, Pb, and Zn in the samples were 0.005, 0.16, 1.0, 15, 0.09, 0.02, and 30 mg/kg, respectively. The highest Cu level was measured in *C. striata* (2.32 mg/kg), and the levels of Ni, Zn, and Mn were highest in *T. trichopterus*, and were 0.23, 48.46, and 61.44 mg/kg, respectively. The highest Cr levels were measured in *P. fasciata* (0.31 mg/kg). The highest Pb levels were measured in *T. microlepis* (0.03 mg/kg). The highest Cd levels were found in *A. testudineus*, *O. niloticus*, *P. brevis*, and *P. proctozystron* (0.007 mg/kg).

Samples of nine fish species, including *Labiobarbus siamensis*, *T. trichopterus*, *T. microlepis*, *Barbonymus altus*, *C. striata*, *A. testudineus*, *P. fasciata*, *P. proctozysron*, and *N. notopterus* were collected at the study sites near Bangpa-in District (Table 3). For overall fish samples collected at this site, the average concentration of Cd, Cr, Cu, Mn, Ni, Pb, and Zn in the samples were 0.014, 0.3, 3, 14, 0.2, 0.03, and 30 mg/kg, respectively. The highest levels of Cu and Cr were measured in *P. fasciata* (7.61 and 0.59 mg/kg, respectively), and levels of Pb, Ni, Zn, and Mn were highest in *T. trichopterus* (0.06, 1.04, 49.29, and 73.29 mg/kg, respectively). The highest level of Cd was measured in *T. microlepis* (0.007 mg/kg).

Table 4 displays the ranges measured for metal concentrations in fish samples. Concentrations of Cd, Cr, and Cu differed significantly between study sites at *p*-value = 0.00, 0.00, and 0.01, respectively. While Cr, Cu, and Mn levels differed between sampling seasons at *p*-value = 0.00, 0.00, 0.03, respectively. In addition, when comparing the level of metals accumulated among fish species, no significant difference were revealed (*p* > 0.05).

### 3.5. Correlation between Heavy Metal Concentrations in Surface Water, Sediment, and Fish Tissue

The correlation between heavy metal concentrations in surface water, sediment, and fish tissue were analyzed using spearman rank correlation. The results exhibited significant positive correlation between Cd in sediment and *T. trichopterus* and *A. testudineus* (*p*-value = 0.013 and 0.042, respectively); Cu in sediment and *P. fasciata* (*p*-value = 0.000); Mn in sediment and *T. trichopterus* (*p*-value = 0.025); Ni in sediment and *T. trichopterus* and *O. niloticus* (*p*-value = 0.000)*;* and Cu in water and sediment (*p*-value = 0.005). Meanwhile, Zn in sediment and *T. microlepis* exhibited significant negative correlation (*p*-value = 0.005).

### 3.6. Human Health Risk Assessment

After calculating the HQ for each heavy metal in each fish species, based on the assumption that the healthy adult inhabitants consumed 21.5 g of each fish species every day, the highest HQs through fish consumption were for Mn, which were 0.2154 and 0.2178 for *T. trichopterus* collected near the industrial estate in Uthai District, and Bangpa-in District, respectively. In terms of the total HQ of Cd, Cr, Cu, Ni, Mn, Pb, and Zn through fish consumption at the study sites near the industrial estate in Uthai District were 0.0261, 0.2894, 0.1164, 0.0201, 0.4921, 0.0460, and 0.4028, respectively (Table 5) and at the study sites near the industrial estate in Bangpa-in District were 0.0582, 0.4879, 0.3265, 0.0683, 0.3909, 0.0606, and 0.4139, respectively (Table 6). Further, the non-cancer risk of Cd, Cr, and Cu which can contribute adverse effects to liver, kidney, and immune system each fish species were expressed as the HI and cumulative HI for all fish species. Of all the studied fish species, *Pristolepis fasciata* caught from Uthai and Bangpa-in Districts showed the highest HI (0.072 and 0.1444, respectively). Meanwhile, the cumulative HI of all fish species from Uthai and Bangpa-in Districts were 0.4319 and 0.8729, respectively. The health risks from exposure to heavy metals from fish consumption either in terms of individual or all fish species consumption in these two areas were within acceptable levels.

## 4. Discussion and Conclusions

As industrialization is one of the factors contributing heavy metal contamination to the environment, flood also plays a significant role in transporting heavy metals, since it can trigger the failure wastewater treatment system in the flooded area [26]. The study, which was conducted following Thailand flooding in 2011, determined the level of heavy metals in surface water, sediment, and fish collected at sites near industrial estates in Uthai District and Bangpa-In District, and showed that heavy metal concentrations in surface water and sediment at sites near industrial estates in both districts did not exceed permissible standards [7,21,22,27]. Moreover, heavy metal levels in surface water and sediments were consistent with the levels reported in previous monitoring studies [28,29,30], with the exception of one sample collected from the area close to industrial estate in Uthai District, which contained higher levels of Mn. It might be explained that although these study sites were flooded during the Thailand flood disaster in 2011, these area did not affected by chemical leakage from industrial estates due to dilution effects from large volumes of floodwater that pass through these areas [31]. In addition, this might be due to the limited effect of flood on polluted, fine-grained sediments flushing in the river reaches with permanently active pollution sources leading to the quick return of the previous state of heavy metal concentration in river system [26].

When comparing the same heavy metal levels in surface water and sediment samples between sites, the sites near industrial estates in Bangpa-in District showed significant higher levels of Cu, Mn, Ni, and Pb in surface water samples, and Ni in sediment samples, than the sites near industrial estates in Uthai District. It might be explained that although the major industries of these industrial estates were similar, the level of heavy metal contamination can be different depending on numbers of factories, pollution control measures, and environmental management effectiveness. In addition, since Bangpa-in district is located downstream of Uthai District, heavy metal input from multiple sources upstream, including agriculture, factories, and communities along the river, can contribute higher heavy metal contamination to the downstream area [32].

The study results also showed that metal concentrations in the fish samples were mostly within acceptable levels [23,24], except for *T. trichopterus* samples, which contained higher Mn levels than the standards set by FAO [24]. *T. trichopterus* is the most abundant species and exhibited the highest level of Pb, Ni, Mn, and Zn. Meanwhile, the average concentration of heavy metals in fishes collected near Industrial estate in Bangpa-in District were comparatively higher than samples from Uthai District, but the difference was not significant. The higher level of heavy metals in fish accord to the higher level of heavy metals in surface water and sediment in Bangpa-in District. However, our study results exhibited lower concentration of all analyzed heavy metals than the study of Sirikanyaporn et al. [33], and lower than fish samples caught from Mun River [34] and Huay Geng Reservoir, Kalasin province [35]. This might be due to the fact that levels of heavy metal in fish can vary with respect to species, feeding behavior, and aquatic environments. When comparing the level of heavy metals among fish categories (carnivorous, herbivorous, and omnivorous) found in this study, heavy metal levels varied. This was inconsistent with several studies that revealed the bioaccumulation was prone to be highest in carnivorous species followed by omnivorous species. In addition, it was also inconsistent to the study of Tanee et al. [35], which found that herbivorous fish showed higher levels of Cu, omnivorous fish showed higher levels of Cd, while Zn and Pb were not significantly different at *p* < 0.05.

In this study, we calculated the non-cancer risk for healthy adult inhabitants corresponds to the fish consumption rate acquired by questionnaire interview. The contribution to total HQ in Uthai District resulted from levels of Mn, Zn, and Cr, respectively, whereas the contribution to total HQ in Bangpa-in District resulting from levels of Cr, Zn, Mn, and Cu, respectively. Regarding the low concentration of metals in fish samples, the HQ and HI values indicated only low levels of risk. However, as some fish species contained higher levels of Mn, we recommend ongoing periodic monitoring of heavy metal concentrations in water, sediment, and aquatic organisms, especially edible species. In addition, the fish consumption rate of children should be investigated for further quantifying the health risk of the children.

## Figures and Tables

**Table 1:** Heavy metal concentrations measured in surface water.

Study Sites	Concentration (µg/L)						
	Cd	Cr	Cu	Mn	Ni	Pb	Zn
Uthai District (*n* = 21)							
range	ND–0.20	0.33–2.50	ND–28.92	47.10–359.10	1.80–55.42	ND–5.50	30.00
mean ± SD	0.2 ± 0.0	0.8 ± 0.9	0.8 ± 0.8 *	300 ± 200 *	8 ± 5 *	1.8 ± 0.0 *	300 ± 0
Median	0.20	0.59	0.53	244.60	5.50	1.80	30.00
Bangpa-in District (*n* = 15)							
range	ND–0.20	14.69–34.87	10.40–28.56	58.92–1029.23	7.51–31.53	5.77–144.60	18.16–163.38
mean ± SD	0.2 ± 0.0	30 ± 5	20 ± 6 *	400 ± 200 *	20 ± 7 *	20 ± 30 *	60 ± 40
Median	0.20	0.88	0.91	63.50	5.50	1.80	30.00
Standards [13]	≤50	≤50	≤100	≤1000	≤100	≤50	≤1000
Detection limits	0.20	0.16	0.28	0.20	1.80	1.80	30

SD = standard deviation; ND = non detectable; * Show significant difference between sites at *p* < 0.05.

**Table 2:** Heavy metal concentrations measured in sediment.

Study Sites	Concentration (mg/kg dw)						
	Cd	Cr	Cu	Mn	Ni	Pb	Zn
Uthai District (*n* = 21)							
range	0.02–0.29	14.69–34.87	10.40–28.56	58.92–1029.23	7.51–31.53	5.77–144.60	18.16–163.38
mean ± SD	0.14 ± 0.08	28 ± 5	22 ± 6	400 ± 200	18 ± 7 *	22 ± 30	60 ± 40
Bangpa-in District (*n* = 15)							
range	0.04–0.28	21.35–36.78	11.81–926.68	164.47–1277.23	10.40–28.00	8.65–22.08	29.57–200.52
mean ± SD	0.16 ± 0.08	31 ± 5	100 ± 300	600 ± 400	21 ± 5 *	16 ± 4	70 ± 40
Standards	<37 ^a^	<300 ^a^	<390 ^b^	<1800 ^a^	<1600 ^a^	<400 ^a^	<960 ^b^
Detection limits	0.02	0.02	0.03	0.02	0.18	0.18	3.0

^a^ National Environmental Board, B.E. 2547 [21]; ^b^ Washington Department of Ecology (WDOE), 1991 [22]; * Show significant difference between sites at *p* < 0.05.

**Table 3:** Distribution of fish species caught in all sampling locations (S1–S12).

Species (*n*)	Category	S1	S2	S3	S4	S5	S6	S7	S8	S9	S10	S11	S12
*Trichopodus microlepis* (100)	Carnivorous	−	+	−	+	+	−	+	−	−	−	+	−
*Trichopodus trichopterus* (140)	Carnivorous	−	+	+	+	+	+	−	−	−	+	+	−
*Puntioplites proctozysron* (5)	Herbivorous	+	−	−	−	−	−	−	+	−	−	−	+
*Notopterus notopterus* (4)	Carnivorous	−	−	−	−	−	+	+	−	−	+	−	+
*Channa striata* (6)	Carnivorous	−	−	−	−	−	+	+	−	−	−	+	−
*Puntius brevis* (3)	Herbivorous	−	−	+	+	+	−	−	−	−	−	−	−
*Barbonymus altus* (12)	Herbivorous	−	−	−	−	−	−	−	+	+	+	+	+
*Oreochromis niloticus* (3)	Herbivorous	−	−	+	−	−	+	+	−	−	−	−	−
*Labiobarbus siamensis* (90)	Herbivorous	−	−	−	−	−	−	−	+	+	+	+	+
*Anabas testudineus* (65)	Omnivorous	−	+	+	−	+	−	−	+	−	−	+	−
*Pristolepis fasciata* (8)	Omnivorous	−	+	−	−	−	−	+	+	−	−	−	−

Remark: *n* = number of fish caught; + Present; − Not present; S1–S7 = Sampling sites in Uthai District; S8–S12 = Sampling sites in Bangpa-in District.

**Table 4:** Heavy metal concentrations measured in fish.

Study Sites	Concentration (mg/kg dw)						
	Cd	Cr	Cu	Mn	Ni	Pb	Zn
Uthai District							
range	0.003–0.007	0.06–0.31	0.36–2.32	2.73–61.44	0.02–0.23	ND–0.03	14.99–48.46
mean ± SD	0.005 ± 0.002 *	0.16 ± 0.07 *	1.0 ± 0.6 *	15 ± 20	0.09 ± 0.06	0.02 ± 0.01	30 ± 10
Bangpa-in District							
range	0.008–0.019	0.12–0.59	0.50–7.61	3.25–73.29	0.05–1.04	ND–0.06	21.05–49.29
mean ± SD	0.014 ± 0.003 *	0.3 ± 0.2 *	3 ± 3 *	14 ± 22	0.2 ± 0.3	0.03 ± 0.02	30 ± 10
Permissible limits	0.5 ^a^	2.0 ^a^	20 ^b^	0.5 ^a^	0.05 ^c^	1 ^b^	100 ^b^
Detection limits	0.001	0.001	0.001	0.001	0.009	0.009	0.15

^b^ MOPH, Thailand [23]; ^a^ FAO 1983 [24]; ^c^ ATSDR, 2005 [25]; * Show significant difference between sites at *p* < 0.05.

**Table 5:** Hazard quotient (HQ) and Total HQ from exposure to heavy metals via fish consumption in the study sites near Industrial estate at Uthai District.

Fish Species	HQ						
	Cd	Cr	Cu	Ni	Mn	Pb	Zn
*Trichopodus trichopterus*	0.0017	0.0113	0.0116	0.0058	0.2154	0.0147	0.0739
*Trichopodus microlepis*	0.0012	0.0267	0.0152	0.0034	0.1533	0.0313	0.0577
*Anabas testudineus*	0.0049	0.0268	0.0127	0.0018	0.0228	-	0.0479
*Pristolepis fasciata*	0.0021	0.0663	0.0036	0.0007	0.0092	-	0.0442
*Channa striata*	0.0028	0.0285	0.0276	0.0012	0.0110	-	0.0255
*Oreochromis niloticus*	0.0038	0.0292	0.0146	0.0025	0.0218	-	0.0253
*Notopterus notopterus*	0.0016	0.0198	0.0063	0.0018	0.0207	-	0.0407
*Puntius brevis*	0.0043	0.0419	0.0148	0.0024	0.0251	-	0.0671
*Puntioplites proctozysron*	0.0037	0.0389	0.0100	0.0005	0.0119	-	0.0205
Total HQ	0.0261	0.2894	0.1164	0.0201	0.4921	0.0460	0.4028

**Table 6:** HQ and Total HQ from exposure to heavy metals via fish consumption in the study sites near Industrial estate at Bangpa-in District.

Fish Species	HQ						
	Cd	Cr	Cu	Ni	Mn	Pb	Zn
*Labiobarbus siamensis*	0.0074	0.0742	0.0366	0.0193	0.0330	0.0133	0.0947
*Trichopodus trichopterus*	0.0033	0.0798	0.0130	0.0286	0.2178	0.0420	0.0647
*Trichopodus microlepis*	0.0067	0.0172	0.0048	0.0017	0.0329	-	0.0251
*Barbonymus altus*	0.0093	0.0619	0.0563	0.0052	0.0277	-	0.0750
*Channa striata*	0.0058	0.0141	0.0045	0.0009	0.0083	-	0.0278
*Anabas testudineus*	0.0055	0.0599	0.0946	0.0023	0.0239	-	0.0308
*Pristolepis fasciata*	0.0056	0.0706	0.0682	0.0028	0.0158	-	0.0303
*Puntioplites proctozysron*	0.0066	0.0484	0.0352	0.0035	0.0148	-	0.0348
*Notopterus notopterus*	0.0080	0.0618	0.0133	0.0040	0.0167	0.0053	0.0307
Total HQ	0.0582	0.4879	0.3265	0.0683	0.3909	0.0606	0.4139
